# Graphene-based cardiac sensors and actuators

**DOI:** 10.3389/fbioe.2023.1168667

**Published:** 2023-05-15

**Authors:** Alex Savchenko, Dmitry Kireev, Rose T. Yin, Igor R. Efimov, Elena Molokanova

**Affiliations:** ^1^ Nanotools Bioscience, La Jolla, CA, United States; ^2^ Microelectronics Research Center, Department of Electrical and Computer Engineering, The University of Texas at Austin, Austin, TX, United States; ^3^ Department of Biomedical Engineering, The George Washington University, Washington, DC, United States; ^4^ Department of Biomedical Engineering, McCormick School of Engineering and Applied Science, Northwestern University, Chicago, IL, United States; ^5^ NeurANO Bioscience, La Jolla, CA, United States

**Keywords:** graphene biosensors, cardiac, graphene, optoelectronics, pacemaker, graphene field electric transistors, graphene electrodes, graphene-mediated optical stimulation

## Abstract

Graphene, a 2D carbon allotrope, is revolutionizing many biomedical applications due to its unique mechanical, electrical, thermal, and optical properties. When bioengineers realized that these properties could dramatically enhance the performance of cardiac sensors and actuators and may offer fundamentally novel technological capabilities, the field exploded with numerous studies developing new graphene-based systems and testing their limits. Here we will review the link between specific properties of graphene and mechanisms of action of cardiac sensors and actuators, analyze the performance of these systems from inaugural studies to the present, and offer future perspectives.

## 1 Introduction

**FIGURE 1:**
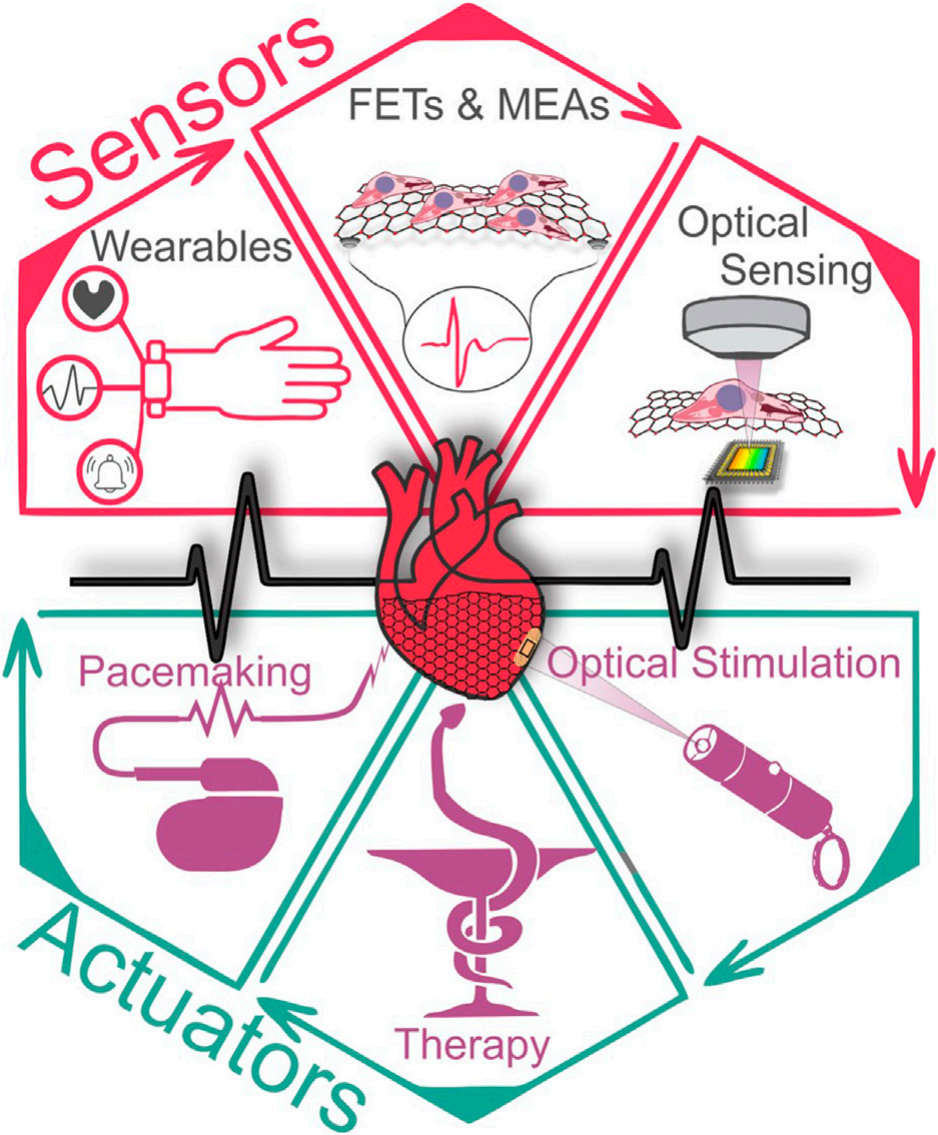
Overview of the graphene-based sensors and actuators for cardiac applications.

The heart is an electrically and mechanically active organ that must work non-stop our entire life, beating on average more than 2.5 × 10^9^ times in human life. We want to ensure that this average will steadily increase for years to come, but today cardiovascular diseases are still the leading cause of death in the world. Due to the life-or-death importance of the heart, scientists have been designing and fabricating diverse bioengineering systems tasked with supporting, monitoring, and modulating the cardiac activity. The development of such systems (Figure 1) faces complex technological challenges because they must satisfy a number of strict requirements: they must conform to the properties of cardiac tissues (e.g., soft, mechanically contracting, electrically active), and demonstrate the long-term reliability, accuracy, and biocompatibility. The fundamental properties of specific materials used as building blocks in these systems impose limitations on what the best achievable performance could be. Therefore, the discovery of a new material with superior properties may open a new era for the development of cardiac sensors and actuators.

Due to the combination of its unique electrical, mechanical, and optical properties, graphene can make it possible to develop superior multi-functional cardiac biosensors and actuators by providing revolutionary technical bioengineering solutions not achievable with other materials (Zhang et al., 2012; Bitounis et al., 2013; Cheng et al., 2017; San Roman et al., 2020; Savchenko et al., 2021). Graphene (Nair et al., 2008; Novoselov et al., 2012), a two-dimensional (2D) carbon crystal, has very a low electrical resistivity, a very high intrinsic electron mobility (Bolotin et al., 2008) (∼200,000 cm^2^/Vs vs. ∼ 1,400 cm^2^/Vs in silicon), and a current density ∼1,000,000 times greater than in copper. The tensile strength of graphene is 130 GPa (vs. 0.4 GPa for structural steel). Graphene has low density (∼2,300 kg/m^3^) and a large Youngs modulus (∼1 TPa). Graphene is the thinnest (0.335 nm), the lightest (0.77 mg/m^2^), and the strongest (42 N/m) material (Lee et al., 2008). Graphene is pliable and stretchable, up to 20% of its initial length. Despite being a 2D material, graphene absorbs a substantial amount (∼2.3%) of incident light (Nair et al., 2008). The absorbance of undoped graphene is independent of the frequency of the electromagnetic radiation (Mak et al., 2008; Nair et al., 2008). As demonstrated in theoretical (Winzer et al., 2010) and experimental (Brida et al., 2013; Tielrooij et al., 2013) studies, graphene exhibits highly efficient multiple hot-carrier generation triggered by the primary photoexcited electron-hole pair (Winzer and Malić, 2012), because carrier–carrier scattering in graphene is more efficient than electron-optical phonon coupling (Iglesias et al., 2015; Massicotte et al., 2021). In this review, we highlight how bioengineers used these properties to develop and fabricate cardiac biosensors and actuators with novel mechanisms of action and advanced technical capabilities.

This review provides a critical perspective on the existing technologies where graphene-based materials enhance the functionalities of cardiac sensors and actuators. Following the introduction, we begin with a broad review of graphene-based sensors functioning either electrically as *in vivo* or *in vitro* cellular interfaces using 1) graphene field effect transistors, 2) and graphene microelectrode arrays, or electrically as 3) wearable electrophysiology sensors and 4) electromechanical pressure sensors, and finally as 5) optical sensors. The third section overviews graphene-based actuators that can function electrically or optically. Finally, we conclude with a section on future perspectives and advances in the upcoming advanced cardiac biosensors and actuators.

## 2 Sensors

The need to continuously, accurately, and reliably monitor various aspects of cardiac activity demands particular properties from cardiac biosensors. The presence of graphene in cardiac biosensors can address many of these demands: electrical properties of graphene offer high conductivity, low impedance, and high signal-to-noise ratio (SNR); mechanical properties of graphene can endow biosensors with flexibility, stretchability, durability, and foldability; a high surface area-to-volume ratio makes graphene-based biosensors extremely sensitive to their environment. Therefore, graphene can offer significant advantages for next-generation biosensors because it can be incorporated into various flexible, electrogenic, lightweight, transparent interfaces and devices for continuous long-term cardiac activity monitoring *in vitro* and *in vivo*.

### 2.1 Graphene field electric transistors

**FIGURE 2:**
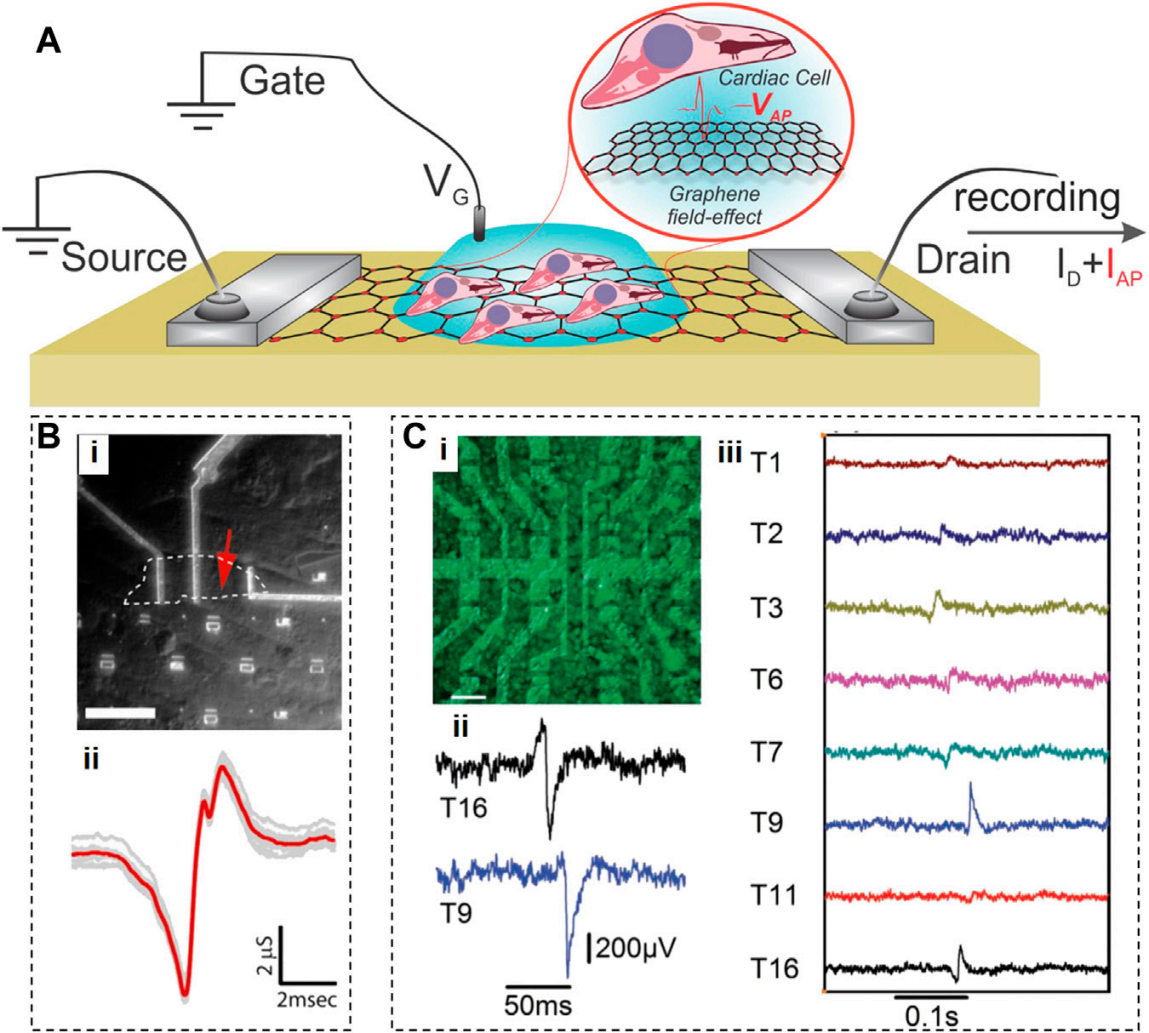
Graphene Field Effect Transistors. **(A)**, Schematic of an electrolyte-gated GFET with cells grown on top of graphene, producing action potential (V_AP_) that translates into the recorded I_AP_ current. **(B)**, Optical photograph of an inaugural GFET (i), and a signal recorded by this GFET from cardiomyocytes. c, Fluorescent image of cardiomyocytes on top of a GFET array (i), extracellular recordings of electrical field potentials by two GFETs (ii), and propagation (iii) of electrical cardiac signals through the cellular layer. Panels reproduced with permission from: **(B)**, (Cohen-Karni et al., 2010), American Chemical Society; **(C)**, (Hess et al., 2011), Wiley-VCH.

Graphene field electric transistors (GFETs) have first been developed in 2004 and have since been fabricated in different shapes and forms (e.g., back-gate, buried gate, etc.), and we would refer the readers to the relevant reviews (Banerjee et al., 2010; Schwierz, 2010; Schwierz, 2013; Chhowalla et al., 2016). The only type of GFETs explicitly suitable for direct interfacing with the cells and monitoring of their electrical activity is the electrolyte-gated EG-GFETs (Zhang et al., 2020). The EG-GFETs are used to measure extracellular field potentials of cardiac cells and tissues (Cohen-Karni et al., 2010; Hess et al., 2011; Kireev et al., 2017a). In a classical GFET, the graphene layer is contacted by two feedlines, and the underlying dielectric controls the channel conductivity as a matter of applied gate potential. In electrochemically-operated GFETs, the feedlines are passivated, and the field effect is applied through a reference electrode immersed in an electrolyte (Figure 2A). The voltage applied through an electrolyte solution builds up an electrical double layer at the interface with graphene, shifting the Fermi level of the graphene layer and changing the device conductance. Due to the extremely high charge carrier mobility in graphene (200,000 cm^2^/Vs8, which is ∼1,000 times higher than in silicon), GFETs can operate at a frequency up to 500 GHz, and detect individual spikes with a high SNR. When cardiac cells are brought in contact with the graphene channel, the electrical signal associated with depolarization of their membrane will affect the local potential distribution and the electrical double layer formed at the graphene surface, resulting in local doping of graphene channel, which is recorded as a change in drain-source current (see Figure 2).

The very first GFETs were fabricated by placing mechanically exfoliated graphene flakes of two different sizes (20.8 μm × 9.8 μm or 2.4 μm × 3.4 μm) on an oxidized Si substrate (Cohen-Karni et al., 2010) (see Figure 2B). These GFETs were interfaced with spontaneously beating embryonic chicken cardiomyocytes cultured on polydimethylsiloxane (PDMS), and recorded some aspects of the cardiomyocyte activity with an SNR of ∼4. Since their signals were of very short duration (1.31 ± 0.04 and 0.76 ± 0.04 ms for GFETs based on larger and smaller graphene flakes, respectively), their origin is not clear. The authors hypothesized that the signals from a larger GFET represent the average of temporarily mismatched extracellular signals from distinct subcellular locations in a single cardiomyocyte, while a smaller GFET provides spatially resolved measurements at the subcellular level. This hypothesis seems doubtful because 1) a spontaneously active cardiomyocyte has an equipotential surface, and 2) 1-ms durations of signals (Figure 2B) acquired by either GFET here was at least 1-2 orders of magnitude shorter than the duration of extracellular field potentials recorded in other studies (Egert et al., 2006; Rastogi et al., 2020a) which means that these GFETs did not perform as expected.

To enable monitoring the activity of multiple cells at once, Hess et al. fabricated an array of 16 GFETs (10 × 20 μm^2^) on sapphire substrates using large-area CVD-grown graphene sheets (Hess et al., 2011). This study cultured cardiomyocyte-like HL-1 cells on GFET arrays, successfully recorded the electrical signals from these cells, and tracked the signal propagation across the cell layer (Figure 2C). The effective gate noise in these GFET arrays indicated that they could detect single voltage spikes from 100 μV with an SNR of 10, indicating a performance similar to microelectrode arrays (MEAs).

To improve the interfacing between soft cardiac tissues and electronic devices, Kireev et al. fabricated GFETs on controllably flexible polyimide-on-steel substrates (Kireev et al., 2017a). These GFETS exhibited Young’s modulus values ∼100 times lower than that of silicon, displayed extremely large transconductance values (up to 11 mS V^−1^), and exceptional charge mobility (over 1,750 cm^2^ V^−1^·s^-1^). These flexible GFETs successfully recorded extracellular action potentials and their spatial propagation across the HL-1 cell layer *in vitro*, as well as electrical signals from primary embryonic rat heart tissues *ex vivo*.

To comprehensively characterize the electrical activity in 3D cardiac cell models, the GFET geometry should preferably also be in a 3D format. Recently, Kalmykov et al. (2019) described the development of 3D self-rolled biosensor arrays (3D-SR-BAs) with GFETs on a pre-stressed metal/polymer support structure for continuous recordings of field potentials from human stem cell–derived cardiac spheroids. The functional test results for 3D-SR-BAs with GFETs were not shown, making it unclear whether this system can perform with high sensitivity and spatiotemporal resolution as envisioned.

### 2.2 Graphene microelectrode arrays

**FIGURE 3:**
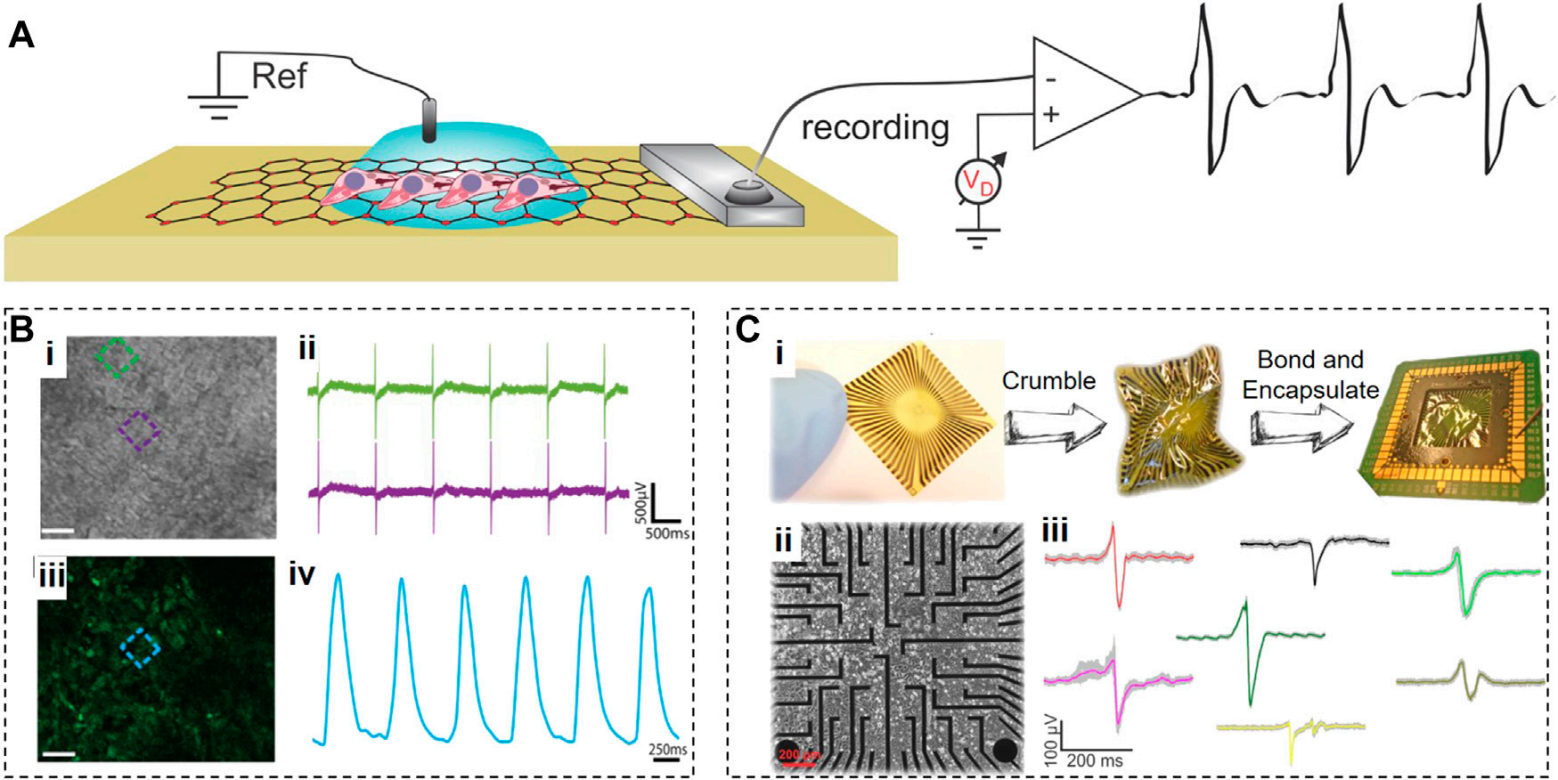
Graphene Microelectrode Arrays. **(A)**, Schematic of an electrolyte-operated GMEA with cells grown on top. **(B)**, Optical (i) and fluorescent (iii) images of cardiomyocytes grown on GMEA array with coincidental electrical (ii) and fluorescent (iv) signals. **(C)**, Flexible GMEAs that crumpled then bonded and encapsulated (i); then used for interfacing with HL-1 cardiac cells (ii) with a variety of APs recorded shown due to differences in cell–chip coupling (iii). Panels reproduced with permission from: **(B)**, (Rastogi et al., 2018), Springer. Panel reproduced from open-access publications: **(C)**, (Kireev et al., 2016), MDPI.

Graphene microelectrode arrays (GMEAs) for monitoring cardiac electrical activity offer several critical advantages, including flexibility, ease of fabrication, high electrical conductance, mechanical and chemical stability, surface roughness, and long-term biocompatibility. The GMEAs typically require one lead per electrode, and a single differential amplifier, with a global reference electrode (Figure 3A). Incorporation of graphene into recording electrodes leads to a significant reduction in their impedance and an increase in their charge injection capacity (Kireev et al., 2017b)*.* The SNR for graphene electrodes is approximately six times lower than for gold electrodes, ensuring the significantly improved sensitivity of graphene-based recording systems (Kuzum et al., 2014; Rastegar et al., 2017).

Rastogi et al. (2018) developed biocompatible transparent 50-µm × 50-µm CVD-synthesized graphene electrodes and coated them with fibronectin for efficient interfacing with fibronectin-conditioned human embryonic stem cell (hESC)-derived cardiomyocytes. The transparency of graphene electrodes allowed parallel optical and electrical recordings to simultaneously detect the changes in the contraction frequency and extracellular field potential durations (Figure 3B).

Kireev et al. (2016) fabricated GMEAs on a flexible polyimide substrate to reduce the mechanical mismatch with soft contracting cardiac tissues (Figure 3C). These GMEAs had 20-µm circular recording apertures and were used to extracellularly monitor the electrical activity from HL-1 cells and acutely isolated embryonic murine heart tissues via electrical impedance spectroscopy. They achieved the SNR of 20 ± 10 for HL-1 cells and 65 ± 15 for heart tissues.

Reducing the dimensions of graphene electrodes can also enhance the integration between electrodes and contracting cardiac tissues. In addition, smaller electrodes would improve the spatial resolution of recordings. However, smaller electrodes usually exhibit high electrochemical impedances and produce recordings of a diminished quality with a lower SNR. This issue was addressed in the study that fabricated transparent 30-µm graphene microelectrodes on float glass substrates followed by electrochemical deposition of the conducting polymer poly(3,4-ethylenedioxythiophene) polystyrene sulfonate (PEDOT:PSS) (Kshirsagar et al., 2018). Graphene/PEDOT:PSS microelectrodes displayed optical transparency in the 55%–90% range with the impedance at 1 kHz ranging from 700 to 50 kΩ. These electrodes successfully recorded the field potentials from contracting cardiomyocytes while simultaneously supporting brightfield optical monitoring of cardiomyocyte contractions.

### 2.3 Silicon nanowire-templated 3D fuzzy graphene platform

**FIGURE 4:**
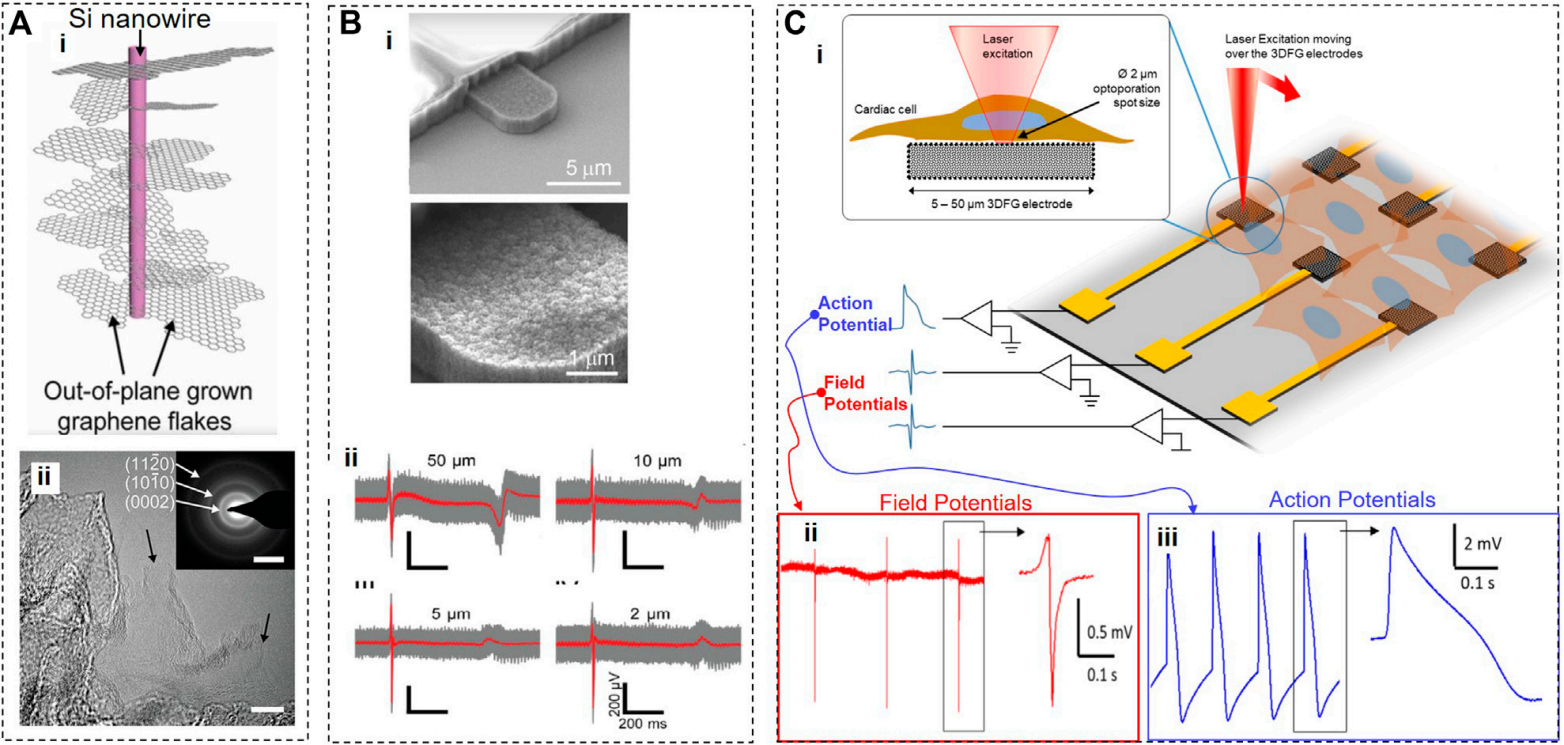
Silicon nanowire-templated out-of-plane synthesized three-dimensional fuzzy graphene. **(A)**, Schematics (i), consisting of a core Si nanowire and surrounding out-of-plane graphene flakes. (ii) HR-TEM image of NT-3DFG with black arrows indicating single-layer graphene flakes (bar, 10 nm). **(B)**, SEM image of NT-3DFG electrodes (ii) and recordings from hESC-cardiomyocytes using 50, 10, 5, and 2 μm electrodes. **(C)**, 3DFG electrode array interfaced with cardiomyocytes, and an ultra-fast NIR laser used to optoporate cells (i). Cardiac field potentials (ii) and action potentials (iii) recorded before and after optoporation, respectively. Panels reproduced with permission from: (Bii), (Rastogi et al., 2020a), Springer. Panels reproduced from open-access publications: **(A)**, (Bondelli et al., 2021), National Academy of Sciences; (Bi) (Dipalo et al., 2021), American Association for the Advancement of Science; (Ci) Carnegie Melon University, College of Engineering; (Cii,iii) (Dipalo et al., 2021), American Association for the Advancement of Science.

Silicon nanowire-templated out-of-plane synthesized three-dimensional fuzzy graphene (NT-3DFG) (Garg et al., 2017) may offer a solution for overcoming the technical challenges of smaller electrodes (Figure 4A). NT-3DFG microelectrodes have a greatly increased overall surface area due to multiple graphene flakes grown in all directions on silicon nanowires, which results in a significant decrease in electrical impedance. For example, 50-μm NT-3DFG microelectrodes exhibited an impedance of 9.4 ± 2.7 kΩ measured at 1 kHz, which is about 140-fold lower than that of Au microelectrodes of the same size (Rastogi et al., 2020a). The impedance of 2-μm NT-3DFG microelectrodes was similar to that of 50-μm Au or 2-μm Pt black microelectrodes. When NT-3DFG microelectrodes of different dimensions (2, 5, 10, and 50 μm) were interfaced with fibronectin-conditioned hESC-cardiomyocytes, they were able to record extracellular field potentials with the amplitude of 400–800 μV and the SNR of about 6. It appears that NT-3DFG electrodes had no noticeable effect on cell viability. The range of durations of extracellular field potentials acquired by NT-3DFG microelectrodes of different dimensions was in the 400–600 ms range, as expected for cardiomyocytes. Notably, the width of the upstroke phase of extracellular field potentials was ∼3 times shorter when recorded by ultra-microelectrodes (2, 5, and 10 μm) compared to 50-μm microelectrodes (see Figure 4B). From a biophysical standpoint, it is unlikely that these differences can be attributed to the averaging of the signals either from multiple locations.

To enable simultaneous recordings from multiple cells, Dipalo et al. (2021) incorporated a silicon nanowire mesh decorated with 3DFG into a MEA platform (Figure 4C). Trains of 8-picosecond pulses of a 1064-nm near-infrared (NIR) laser of high intensities (414–2419 W/mm^2^) and high repetition rate (80 MHz) were used for transient poration of cell membranes of hiPSC-derived cardiomyocytes cultured on 3DFG electrodes. Authors hypothesized that 3DFG-assisted membrane poration was produced by an electron plasma generated in graphene by a laser but did not verify this hypothesis experimentally. Based on numerous published studies, a more likely explanation for observed optoporation is that a high-intensity high-repetition pulsed NIR laser can cause the poration of the cell membrane on its own without any help from graphene (Davis et al., 2013; Xiong et al., 2016; Bondelli et al., 2021). In this study, a 2-µm laser spot illuminated both 3DFG (∼0.5 µm in height) and cardiomyocytes, which affected the fluidity and integrity of the cell membrane, allowing its tighter adhesion to numerous out-of-plane graphene flakes from 3DFG electrodes and leading to the formation of pores. The viability of cardiomyocytes was excellent before a laser-triggered poration. A relatively short recording time (few minutes) after optoporation raises a possibility that processes leading to membrane poration and/or poration itself may affect the viability of cardiomyocytes. However, this study demonstrated that optoporation could be repeated several times on the same cell, supporting the notion of low invasiveness of this method. Although the authors dismissed the possibility of thermal effects in laser-illuminated 3DFG, it should be taken into account that their inaugural study with a 3DFG platform (Rastogi et al., 2020b) demonstrated that light with intensities 100 times lower than used by Dipalo et al. (2021) led to a substantial increase in extracellular temperatures (up to 7°C) resulting in photothermal activation of cells.

Optoporation provided transient access for the 3DFG MEA platform to an intracellular compartment of cardiomyocytes and allowed the recordings from the inside of a cardiomyocyte with an SNR up to 43 dB. The shape of these signals resembled the intracellular action potentials recorded using a patch clamp method with a few notable exceptions. In intracellular signals acquired by 3DFG MEAs, the amplitude of a depolarizing component is comparable to that of an afterhyperpolarization component, and the average overall amplitude was ∼ only 3.56 ± 1.96 mV. In addition, there is significant variability in 3DFG-acquired intracellular signals (e.g., 10-fold differences in the signal amplitude) due to drastically varied levels of coupling reached after optoporation. Further, the different areas of the same reasonably large 3DFG electrode may be simultaneously exposed to an extracellular environment and have partial access to intracellular compartments of a cardiomyocyte, producing variable signals of a hybrid identity. Technological development of the 3DFG MEA platform may address these issues in the future.

### 2.3 Wearable graphene electrodes for electrophysiological recordings

**FIGURE 5:**
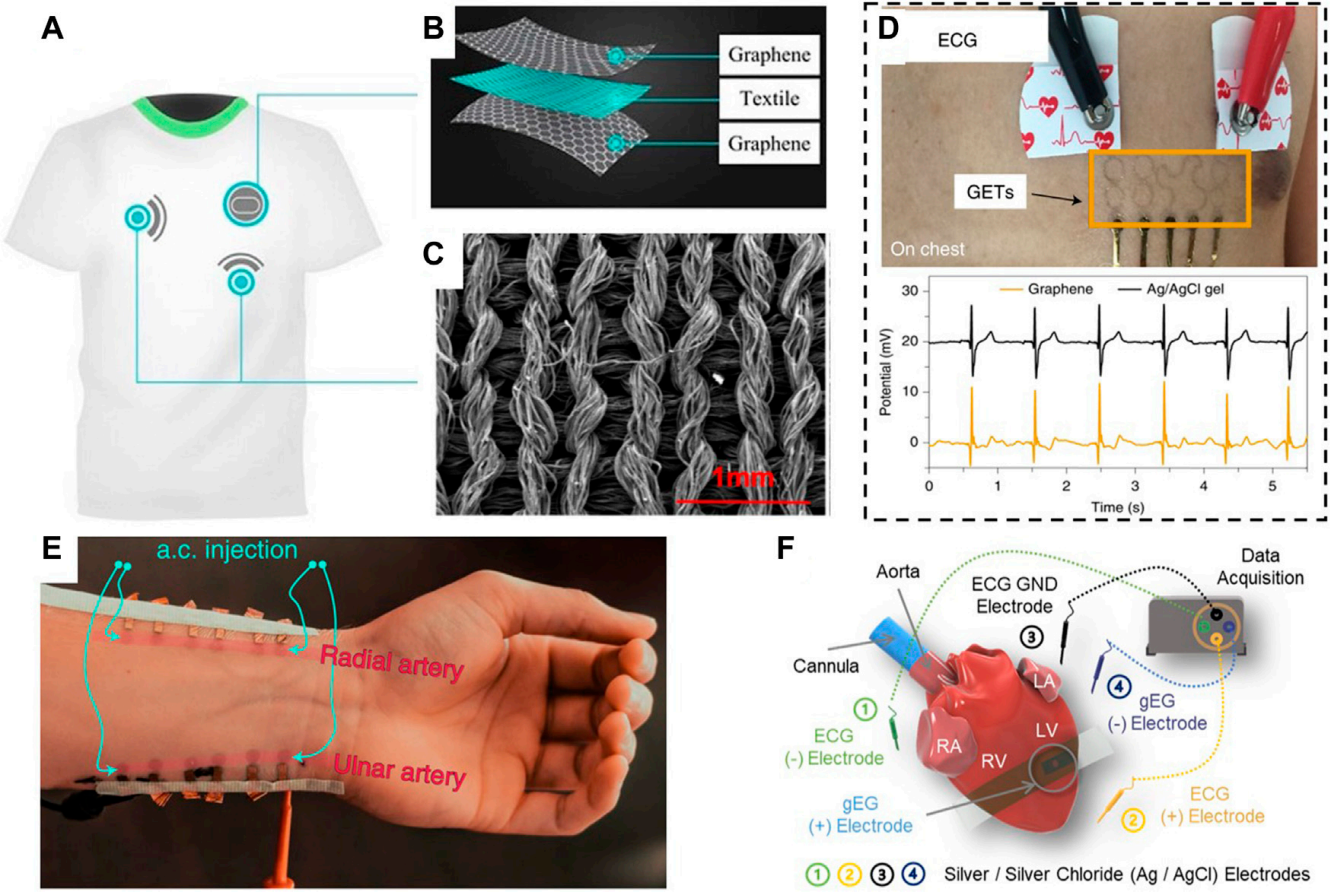
Overview of wearable applications of graphene for cardiac electrophysiology. **(A,B)**, Graphene-based textiles can be embedded into clothes (i.e., a shirt) or wristbands for ECG or pulse monitoring. **(C)**, SEM image showing the morphology of the graphene morphed with textiles. **(D)**, Optical image and exemplary ECG time trace recorded using graphene electronic tattoos. **(E)**, Example of GET arrays placed on the subject’s wrist and used for continuous blood pressure monitoring. **(F)**, Schematic of graphene biointerface electrodes placed right on the heart for direct pacemaking. Panels reproduced with permission from: **(C)**, (Yang et al., 2018). American Chemical Society; **(D)**, (Kireev et al., 2021), Springer Nature Ltd.; **(E)**, (Kireev et al., 2022b), Springer Nature Ltd. Panels reproduced from open-access publications: **(A,B)**, (Lou et al., 2016), MDPI; **(F)**, (Lin et al., 2022), Wiley.

An electrocardiogram (ECG) is a vital physiological measurement routinely performed for the evaluation of the cardiac activity. ECG time trace (see example in Figure 5D) contains rich information about the heart’s function and health. From observing the ECG waveform, trained medical practitioners (and trained ML algorithms) can suspect a myocardial infection, arrhythmia, tachycardia, ischemia, infarction, and other abnormalities. Holter monitor is a portable device capable of long-term monitoring of ECG, yet it is commonly bulky, uses uncomfortable gel electrodes, and there is always a trend to create more user-friendly wearable technological solutions. The most common ECG electrodes require adhesives or conductive gels, but these wet electrodes are unsuitable for long-term monitoring and can become uncomfortable. Textile-based dry, flexible, highly conductive wearable EEG graphene electrodes can simultaneously provide improved comfort and the ability to record high-fidelity electrocardiograph signals (Batchelor and Casson, 2015; Yapici and Alkhidir, 2017; Hallfors et al., 2018) (Figures 5A, B).

Such hybrid electrodes are flexible, exhibit strong electrical performance, and can record ECG signals for a week without degradation in the signal quality (Lou et al., 2016). Integration of graphene nanomaterials with textiles allows for robust electrophysiological sensing regardless of physical movement and stretching imposed on the graphene-textile structure (Figure 5C). Graphene-based e-textiles can be inkjet-printed for wearable electronics that monitor ECG signals, heart rate, and heart rate variability (Karim et al., 2017). Remarkably, users can undergo regular activities, such as taking a shower or running 20 miles, without risking detachment of inkjet-printed electrodes. All textile armbands were shown to record high-fidelity signals from a single arm that match ECG signals recorded by conventional gel-based electrodes (Acar et al., 2018).

Fabrication of a robust bendable conductive layer on a porous textile surface could be technically challenging. Karim et al. (2017) solved this problem by printing an organic nanoparticle-based surface onto textiles to create a background hydrophobic breathable coating. Subsequent inkjet printing of a continuous conductive electrical path onto this background resulted in graphene-based printed e-textiles with reduced sheet resistance. These e-textiles successfully performed heart rate monitoring in healthy male volunteers. The washability test of graphene e-textiles revealed that they could withstand ten home laundry washing cycles, although the fabric resistance increased successively with each washing cycle.

Another study developed and compared three types of graphene-based dry flexible ECG electrodes constructed by 1) vacuum-controlled absorption of graphene layers on the top and bottom surfaces of flexible polyester fibers, 2) using a 60-µm-thick graphene paper, or 3) depositing few-nanometer-thick graphene films on 280-µm-thick polyethylene terephthalate substrates (Lou et al., 2016). The intrinsic internal impedance was the lowest in graphene-paper electrodes (19 Ω) and the highest in graphene-textile electrodes (2.9 MΩ). When testing wearable graphene textile electrodes on healthy volunteers for 1 week, this study discovered that graphene electrodes were able to acquire typical human ECG signals with a high SNR (up to 32 dB) in different states of motion and without any decrease in the signal quality over time.

The majority of the above-mentioned wearable devices feature bulky substrates that do not adhere well to the skin. This problem was recently solved by creating the so-called graphene electronic tattoos (GETs) designed as filamentary serpentines (Kabiri Ameri et al., 2017; Kireev et al., 2021). GETs are composed from a high-quality electronic-grade CVD-grown large-scale graphene monolayer supported by a ∼200-nm-thick transparent thermoplastic Poly(methyl methacrylate) (PMMA) substrate. GETs have a total thickness of 463 ± 30 nm, an optical transparency of ∼85%, and a stretchability of ∼40% which means that they are ultrathin, flexible, transparent, and lightweight and can conform to skin morphology and deformations. GETs are fabricated by a cost- and time-effective “wet transfer, dry patterning” method, providing a “high-end, low-cost” solution for ECG monitoring. They are superior wearable systems and can be placed either on the chest or two hands of a subject to reliably detect ECG signals, while being entirely imperceptible (Figure 5D). The GET-skin interface impedance is on par with ECG standard silver/silver-chloride (Ag/AgCl) gel electrodes while offering superior comfort, mobility, and reliability. Besides classic electrophysiology, the GETs can be placed on top of a wrist, and, by injective a.c. signal into the arteries, and measuring a.c. change in the voltage (method known as Bioimpedance), it is possible to extract Blood Pressure (Figure 5E).

The increase in the technical complexity of GETs may allow their use for recording of electrical signals directly from the surface of the heart (Figure 5F). To achieve this goal, previously described GETs were modified to include additional components such as two transparent and flexible silicon elastomer support layers with an electrode opening of 1, 1.5, 2, or 3 mm, and a flexible adhesive ultrathin (10-µm) conductive gold tape to connect GET with the data acquisition and stimulation hardware (Lin et al., 2022). Three-layer GETs with smaller electrode openings provided the most reproducible qualities, including the lowest sheet resistance and lower interface impedance. The average value of area-normalized impedance at 1 kHz for 1-mm opening 3L GETs is on par and even exceeds that value of highly conducting PtTe_2_ and gold e-tattoo. These GET-electrodes were successfully applied for monitoring cardiac electrical activity in an *ex vivo* Langendorff-perfused mouse heart model. The simultaneously recorded ECG and graphene electrogram show a good temporal correlation between R-waves and elapsed time between two successive R-waves, and the SNR above 20 for GET-electrodes with a 1-mm electrode opening.

To improve the spatial resolution of the cardiac electrical mapping, a novel array of micropatterned GETs (mGETs) was fabricated (Lin et al., 2022) where the layer of graphene was patterned into multiple feedlines and individually addressed; an extended PMMA substrate was providing the support at the bottom; and the top passivated PMMA layer only had openings at the contact and electrode opening sites. The architecture of this PMMA-graphene-PMMA device is similar to classic MEAs, while offering a thickness below 500 nm. The 2 × 2 mGET-arrays were placed on an *in vivo* beating rat heart and recorded electrical signals from both the right and left ventricles.

### 2.4 Wearable pressure sensors

Measuring blood pressure within a human body is critical for continuous monitoring of health conditions, allowing timely diagnosis of potential dysfunction of the cardiovascular system. Wearable pressure sensors must exhibit a broad dynamic range, high sensitivity, linearity, rapid response, durability, stretchability, and reproducibility (Pang et al., 2018; Xia et al., 2018; Huang et al., 2019; Ray et al., 2019; Yang et al., 2019). Due to its exceptional mechanical and electrical properties, flexibility, and biocompatibility, graphene can easily satisfy these requirements. Critically, the sensitivity of graphene-based pressure sensors can be further increased by incorporating such structures as aerogels, textiles, fiber yarns, fish-scale-like structures, woven fabrics, films, spring-like mesh, grid patterns, and porous structure/foams.

Piezoresistive graphene-based sensors (Cao et al., 2021) represent the most common pressure sensor. These sensors experience the change in electrical resistance of a material when stretched (Zheng et al., 2020), and have significant technical advantages such as relatively low cost, ease of fabrication, high sensitivity, large detection range, enhanced durability, low power consumption, and facile signal read-out (Chen et al., 2018).

Du et al. (2016) developed a wearable piezoresistive sensor (GNWF) by dipping a non-woven fabric into a GO solution and reducing a deposited GO layer *in situ* using hydriodic acid. The fabrication cost of a sensor directly integrated into a fabric is expected to be minimal, which can lower the barriers to future commercialization. The performance of GNWF sensors was strongly dependent on the loading of rGO and the strain strength. For example, GNWF sensors with the rGO loading in the 0.7–1.5 wt% range acted as negative pressure sensors at a small strain range (<5%) but became positive pressure sensors at higher strains. On the other hand, in the GNWF sensors with the 2.3 wt% rGO loading, the ΔR/R0 rations were always positive. The authors hypothesized that, at small strains (<5%), the resistance of the GNWF sensors containing low rGO concentrations is decreasing due to the formation of additional conductive paths, but at higher strains, fibers in GNWF sensors became disentangled and disconnected, resulting in the increased resistance. Critically, at larger strains (>10%) (when ΔR/R0 becomes more positive), the GNWF sensors experienced destructive deformation, leading to irreversible sensor deterioration. The best-preforming GNWF sensors exhibited a gauge factor of −7.1 at 1% strain, and a sensitivity of 0.057 kPa^−1^. When tested on human volunteers, the GNWF sensors were responsive to stretching, bending, pressure, and respiration, but these measurements were qualitative rather than quantitative.

Yang et al. (2017) presented a piezoresistive pressure sensor based on graphene woven fabrics (GWFs) with a crisscross structure positioned on a 100-μm thick elastic PDMS film. This sensor exhibited an extremely high gauge factor of 500 (0%–2%), 10^3^ (2%–6%), and 10^6^ (>8%) due to the unique crisscross morphology of GWFs and crack propagation mode. Searching for the acceptable compromise between the sensor’s sensitivity and the linearity of its responses, this study determined the optimal balance of these two parameters using the PDMS substrates with Young’s modulus of a few MPa, which provided the linearity of ∼0.98, and a formidable sensitivity (gauge factor of ∼12). The utility of this sensor for cardiovascular monitoring under different experimental conditions was successfully demonstrated on a healthy volunteer before and after high-intensity exercise.

Xia et al. (2018) demonstrated a 3D pressure sensor consisting of continuous 3-D graphene films with closely packed graphene hexagonal concentric nanoribbon rings. These 3-D hybrid graphene structures were supported and protected by hierarchical structured PDMS films molded on *Epipremnum aureum* leaves to achieve the microstructured surface with convex polygonal structures approximately 4 μm in height. This sensor exhibited a high sensitivity of 110 kPa^−1^, a wide pressure working range of up to 75 kPa, a low detection limit (0.2 Pa), a fast response time (<30 ms), high stability for more than 10,000 loading/unloading cycles, and excellent durability without exhibiting hysteresis. This sensor was shown to accurately detect the pulse at rest and after an exercise and to determine the systolic and diastolic blood pressure, the ventricular rate, and the heart rate from the pulse waveform.

Ai et al. (2018) fabricated a piezoresistive pressure sensor by sandwiching 5-μm polystyrene (PS) micro balls coated with rGO between two thin, flexible electrode-attached PDMS films. In this sensor, a small compression leads to more contacts of micro balls with each other and electrodes, resulting in additional conductive pathways and a decrease in the device’s resistance. This study demonstrated that this sensor has a high sensitivity of 50.9 kPa^−1^ at 3–1,000 Pa, a detection limit of ∼3 Pa, fast response time—50 ms at a fast-pressing frequency of 5 Hz, high durability at over 20,000 loading-unloading cycles, and low energy consumption of ∼1 μW at a low bias voltage of 1 V. Used by a volunteer, this sensor successfully detected a typical radial artery pulse waveform with three clearly distinguishable peaks in one cycle pulse, showing that it can be used for continuous and accurate detection of cardiovascular parameters.

**FIGURE 6:**
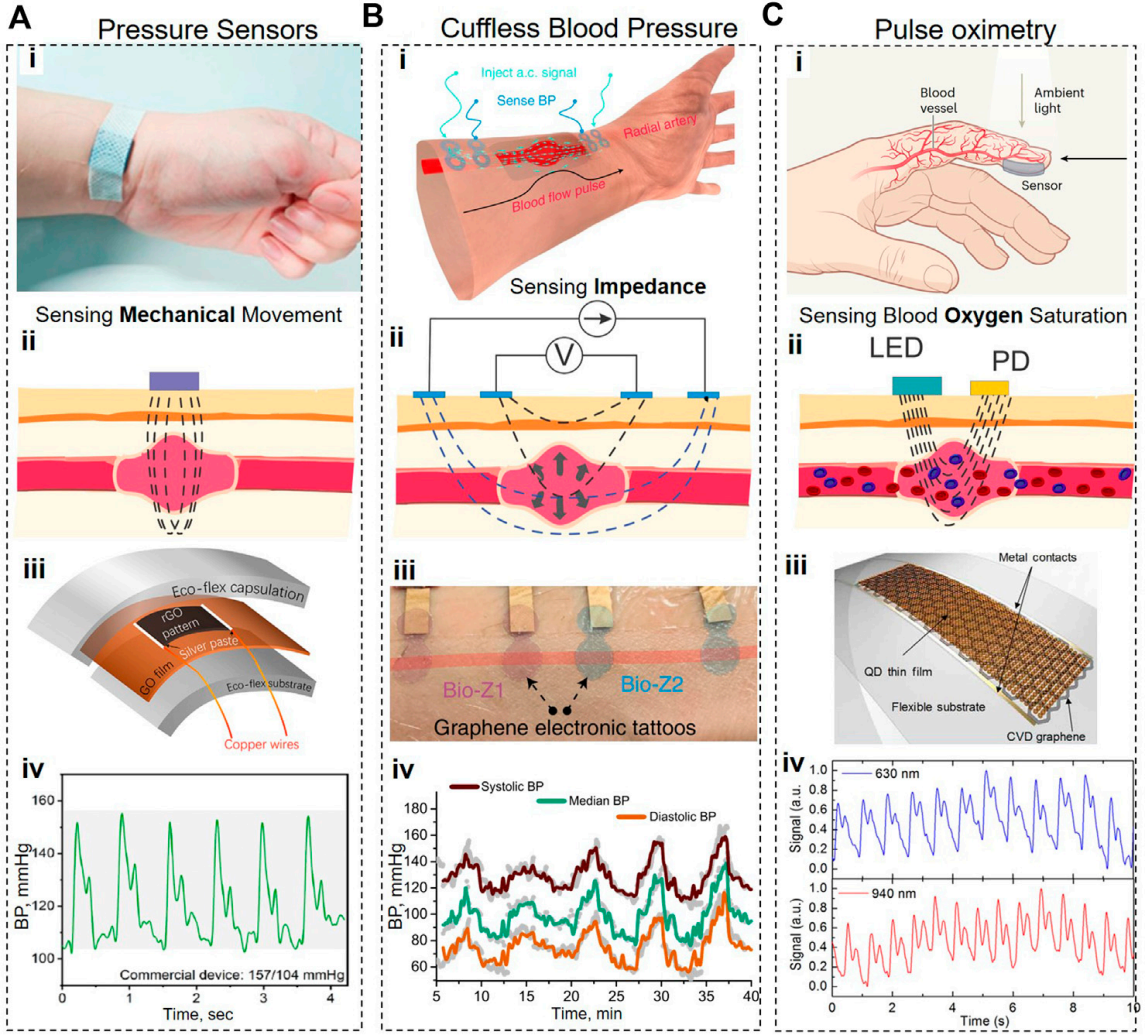
Graphene-based cardiovascular wearable systems. **(A)**, Piezoresistive pulse measurement system. **(B)**, Schematic and working principle of Bioimpedance-based Blood Pressure monitoring system. **(C)**, Schematics and working principle of PPG-based pulse oximetry system. In all cases, (i) shows the photograph or 3D representation of the device, (ii) shows the operation principle, (iii) shows the schematic or optical images of the devices featuring graphene nanomaterials, and (iv) features the recorded timetraces. Panels reproduced with permission from: **(A)**, (Wu et al., 2020), American Chemical Society; **(B)**, (Kireev et al., 2022b), Springer Nature; **(Ci)**, (Akinwande and Kireev, 2019). Springer Nature. Panels reproduced from open-access publications: **(Ciii,iv)**, (Polat et al., 2019), American Association for the Advancement of Science.

Wu et al. (2020) (Peng et al., 2021) developed a pressure sensor consisting of the GO film with pressure-sensing patterns containing laser-scribed graphene (LSG) and two encapsulating layers of Eco-flex (Figure 6A). The authors state that “due to the advantage of the laser scribing process, the sensors could be manufactured in arrays with customized patterns and then cut into small independent units, which is beneficial for low-cost and mass production.” In contrast to the majority of graphene-based pressure sensors, this device is a positive pressure sensor because it exhibits an increase in resistance in response to the increase in external pressure. It appears that the positive piezoresistive property of this sensor comes from the fact that pressure in the physiological range is insufficient to produce noticeable changes in interlayer spacing and conductivity in LSG. Instead, under pressure, this sensor underwent more extensive stretching in the XY plane than compression deformations along the Z axis. This sensor exhibited the exponential relationship between the changes in resistance and pressure applied along the Z-axis, which means that it can amplify electrical signals by utilizing external pressure. During the optimization studies, the authors varied a laser scribing angle from 0° to 90° and discovered that the sensor’s sensitivity is also positively correlated with the scribing angle: e.g., at 200 kPa, the sensitivity of the 0° sensor was 17 kPa^−1^ vs. 434 kPa^−1^ for the 90° sensor, while the ΔR/R0 of the 0° sensor was∼ 1,200%, vs. ∼16,500% for the 90° sensor. This study also found that finer graphene stripe patterns offer a higher sensitivity due to defects. Therefore, the 90° sensors with narrow graphene patterns exhibited ultra-high sensitivity, reaching the sensitivity of ∼360,000% at ∼ 195 kPa. Using this sensor, the authors performed real-time monitoring of the pulse waveform, which allowed them to infer multiple parameters (e.g., blood pressure, viscosity, velocity, and the resistance of blood flow) crucial for the evaluation of cardiovascular health.

Peng et al. (2021) fabricated a flexible pressure sensor by ink-printing GO onto electroless nickel and immersion gold electrodes, subjecting the GO ink to a foaming procedure, and, then, reducing it *in situ*. Subsequently, this pressure sensing element was encapsulated into a polyethylene terephthalate/polyethylene vinyl acetate (PET/EVA)-laminated plastic film to mitigate such problems as the inability of porous graphene to withstand shear forces. This manufacturing process can be fully automated, potentially allowing a low-cost fabrication. When pressure is applied to porous graphene, its deformation leads to an increase in the overlapping areas of compressed pores and a subsequent decrease in the electrical resistance of this device. This porous graphene-based sensor exhibited excellent sensitivity (53.99/MPa), high resolution (<0.3 kPa), broad range (0.3 kPa–1 MPa), and impressive repeatability (1,000 cycles). The authors demonstrated that this sensor was able to accurately monitor several vital cardiovascular parameters, including heart rate, respiration rate, and blood pressure.

Shirhatti et al. (2021) developed a multifunctional flexible wearable sensor using a percolative layer of 2-D microwave-exfoliated GO nanosheets on laser-patterned gold interdigitated electrodes (IDE). This graphene on laser-patterned electrodes (GLE) sensor was fabricated on PET and/or substrate and then overcoated with another PDMS layer for protection from external factors. According to this study, the primary sensing mechanism of the GLE sensor for strains up to ∼1,000 με is based on the disruption of conductive paths formed by graphene nanosheets between different regions of the IDE. To achieve high sensitivity, this mechanism requires a very thin (but still discernible) layer of graphene nanosheets because when the number of graphene sheets increases, it leads to an increase in the number of conductive paths, resulting in a smaller ΔR/R0 ratio in response to applied pressure. Due to the ultrathin sensing layer and the circular sensor design, GLE sensors can offer omnidirectional strain detection and exhibit exceptionally high sensitivity. Indeed, these sensors were able to achieve an exceptional strain resolution (better than 0.02%), and very high gauge factors (up 6.3 × to 10^7^). This study demonstrated that a single GLE sensor can be used for monitoring the heart rate, breathing rate, limb movements, body temperature, or body dehydration. This feature raises the possibility that changes in one parameter can affect the detection accuracy of another parameter. Indeed, these sensors were found to be very susceptible to movement noise/artifacts.

Luo et al. (2021) presented a flexible pressure sensor with a pyramid-structured 500-nm graphene nanowalls (GNWs) electrode and a hybrid conformal 1.5-μm thick PDMS dielectric layer with an 80-nm thick sputtered zinc oxide (ZnO) film (Microconformal GNWs/PDMS/ZnO electrode-dielectric integration (MEDI). Due to the MEDI design, the polarized electric field caused by piezoelectric effects of the ZnO layer can greatly enhance the capacitance of the sensor and significantly increase the piezo-capacitive effects upon deformation, resulting in improved sensitivity and the pressure-response range. This piezocapacitive sensor exhibited an ultra-high sensitivity (up to 22.3 kPa^−1^), a fast response time (25 ms), and a broad pressure range (22 kPa).

Zaretski et al. (2016) fabricated a novel piezoresistive sensor by depositing closely-packed self-assembled metal (gold, silver, or palladium) nanoislands onto a supporting graphene layer. These sensors exhibited non-linear changes in resistance versus strain with at least two inflection points, which potentially indicated different sensing modes. In the lowest strain regime (0.001%), the piezoresistive effect is most likely due to the changes in tunneling current when the nanoislands underwent small changes in separation. With the increase in strain, cracks start to appear in the nanoisland film and graphene layer, leading to an increase in the distance between the nanoislands and a consequent decrease in the conductivity. The sensitivity of a sensor with palladium nanoislands on graphene is among the highest of any thin-film strain sensor with the gauge factor at 1% strain being 1,335. When used for pulse monitoring, this sensor accurately resolved the pulse waveform with clearly distinguishable systolic and diastolic pressures, the dicrotic notch (aortic valve closure), and other cardiac cycle events. To ensure biocompatibility, this study used the sensors with gold nanoislands for detecting the contractions of stem cell-derived cardiomyocytes, and determined that these sensors exhibited sub-millisecond response time, and very high signal-to-noise ratio (between 42 and 100 for CM contractions of different strength). The amplitude and the temporal profile of CM contractions, as detected by our sensor allow a detailed characterization of CM response and enable testing of various pharmacological compounds for drug discovery applications.

In addition to harnessing piezo effects for pressure monitoring, the changes in blood pressure can also be detected using a bioimpedance (Bio-Z) method. This method involves injecting a low amplitude high-frequency current via two outer electrodes and measuring the resultant change in voltage via two inner electrodes (Sel et al., 2019) (Figure 6B). When placed over arteries and in larger arrays, the Bio-Z can be used to record blood pressure; purely non-invasively and without applying any stress or discomfort to the patients. The recent study found that GETs are the most suitable wearable systems since they afford tight and non-shifting contact with the skin, yielding superior measurement reproducibility (Kireev et al., 2022b). Combination of Bio-Z with GETs resulted in improved measurement accuracy (0.2 ± 5.8 and 0.2 ± 4.5 mmHg, for SBP and DBP, respectively), on par with the Grade-A of blood pressure monitoring systems (Kireev et al., 2022b), demonstrating a novel solution for continuous cuffless BP tracking. The significant advantage of GETs for this task is their intimate conformal contact with the skin (see Figure 6B), requiring only a primary calibration and subsequent continuous usage. Such system, unlike the cuff-based solutions, is actually capable of nocturnal operation, when high fidelity is required without disturbing patients (Kwon et al., 2020; Gaffey et al., 2021).

### 2.5 Optical sensors

Monitoring of heart rate, arterial oxygen saturation, and respiratory rate can be performed using photoplethysmography. This non-invasive method requires sending a light signal through the skin and optically detecting the changes in that signal caused by the contraction/expansion of blood vessels during the cardiac cycle. The recent study (Polat et al., 2019) introduced a new class of flexible and transparent wearable photoplethysmographic devices from graphene sensitized with quantum dots (GQD) (Konstantatos et al., 2012). In GQD photodetectors, graphene serves as an ultrathin large-area flexible transparent layer with exceptional electronic mobility, while a 30-nm layer of PbS quantum dots is tasked with absorbing the light modulated by changes in the volume of blood vessels (Figure 6C). GQD photodetectors are semitransparent (maximum recorded absorbance is 25% at 633 nm), and can operate either in a reflectance or transmission mode: the light signal to be modulated during the cardiac cycle comes either from a green LED (535 nm, 0.4 mW) integrated into a device (a reflectance mode) or from the ambient light (a transmission mode). Due to their broadband absorption, GQD photodetectors can use solely ambient light, which allows a wearable device to detect vital signs that require continuous tracking over a long time.

**FIGURE 7:**
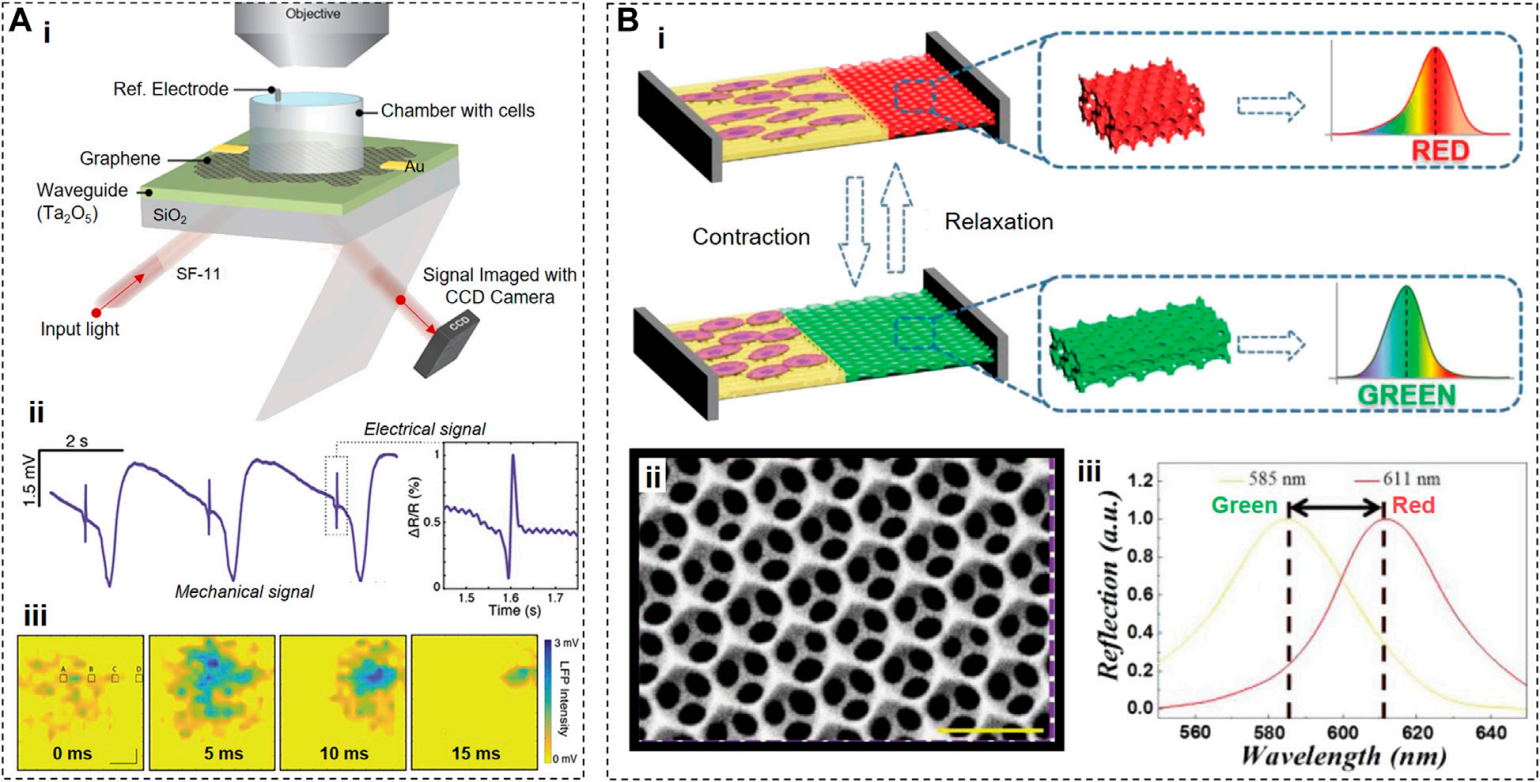
Optical Sensors. **(A)**, Working principle of a CAGE sensor (i) that can be used to reconstruct the electrical and mechanical activities (ii,iii) of cardiac cells that locally shift the Fermi level of graphene due to electrical membrane depolarization. **(B)**, Working principle of color shifters (i), where graphene based nanostructures (ii) can change their color upon mechanical contraction of the cardiac cells (iii). Panels reproduced with permission from: **(A)**, (Balch et al., 2021), American Chemical Society; **(B)**, (Li et al., 2020), Wiley-VCH.

To enable label-free monitoring the activity of cardiac cells and heart tissues, Balch et al. developed a critically coupled waveguide-amplified graphene electric field sensor (CAGE sensor) (Balch et al., 2021) (Figure 7Ai). The CAGE sensor takes advantage of the fact that, in the mid-IR region, the electrical field can modulate the light absorption in graphene by shifting the Fermi level (Wang et al., 2008). Electrogenic cells, including cardiomyocytes, generate the extracellular electrical field, and that field can act as a local electrostatic gate and dynamically modulate the light absorption of a graphene substrate directly under a cell. The changes in light absorption can lead to changes in light reflectivity and provide information about cellular activity.

Since voltage-driven changes in light absorption of graphene are relatively small (∼1% per 200 mV) (Wang et al., 2008), the authors designed a sophisticated hybrid sensor to amplify these changes. Specifically, the CAGE sensor includes a prism-coupled slab waveguide coated on one side by large-area monolayer graphene, a coupling layer of 1,000 nm SiO_2_, and a waveguide layer of 150 nm Ta_2_O_5_ deposited on one face of a glass prism. This configuration allowed the light signals to be reflected (and, thus, amplified) ∼100 times before reaching a CCD camera, resulting in 0.07% relative change in reflectivity under 100 µV voltage modulations with the SNR of ∼5.

The proof-of-concept experiments demonstrated that, by monitoring the reflectivity changes in a graphene cell substrate, the CAGE sensor acquired a complex multicomponent signal from primary chicken cardiac tissues (Figure 7Aii). The major component (∼90%) of this signal is mechanical in nature and represents the contractile activity, while the minor component was described as “electrical.” The CAGE sensor detected the contractile activity because such activity can modulate the local refractive index due to the change of the contact surface area between cardiomyocytes and a graphene substrate during contractions which inevitably led to the reflectivity changes. Suppose the minor component indeed represents the electrical activity of cardiomyocytes. In that case, it is not clear 1) why, in some cases, the CAGE “electrical” signals precede the contractile activity when in cardiomyocytes, the electrical activity always starts before the contraction initiation (Figure 7Aii), or 2) why the duration of transient “electrical” signals detected by the CAGE sensor is significantly shorter than the duration of cardiac action potentials (Figure 7Aii, iii). Further studies and improvements may be able to resolve these inconsistencies.

To visualize the contractions of cardiomyocytes by observing the color shift of cell substrates, another group (Li et al., 2020) developed a hybrid anisotropic film containing low-adhesion polyethylene glycol diacrylate (PEGDA) and high-adhesion reduced graphene oxide (rGO)-doped gelatin methacryloyl (GelMA) (Figure 7B). By etching previously deposited 320-nm silica nanoparticles, the PEDGA area was transformed into an inverse opal hydrogel scaffold with orderly periodic micro–nanostructure that blocked the transmission of light waves of a specific wavelength. rGO was incorporated into this sensor to increase contrast and improve cell adhesion and beating frequency of cardiomyocytes by endowing GelMA hydrogels with conductive properties. Cardiomyocytes were preferentially located on rGO-doped GelMA, and, during contractions, stretched the lattice of the inverse opal PEGDA area. As a result, the dimensions of microstructures in the PEGDA area also changed, causing the spectral reflection shift from 610 to 560 nm (i.e., from red to green). Since the Young’s modulus and stretch ratio of the PEGDA area are known, this sensor allows the calculations of the contraction force based on the shift of the reflection peak.

## 3 Actuators

Contrary to biosensors, actuators are designed to intervene with the existing cardiac activity when correcting cardiac functional deficiencies is needed. Actuators can initiate and restore cardiac contractions (e.g., defibrillators) or support the heart beating at a normal rate (e.g., pacemakers). To allow repeated use, cardiac cell stimulation technologies must be minimally invasive, rely on physiologically relevant mechanisms, and enable fast and reversible changes in the cell membrane potential.

There are two fundamentally distinct types of graphene-based actuators: electrical and optoelectronic. During electrical stimulation, graphene electrodes deliver electrical currents from an external current generator to cardiac cells and tissues, while during optical simulation, cells are stimulated by the electrons generated by light directly in graphene.

### 3.1 Electrical stimulation

Electrical stimulation of cells can be beneficial both in *in vitro* applications (e.g., when patient-specific iPSC-derived cardiomyocytes can be paced at different frequencies to evaluate their functional properties and effects of personalized drugs) and in *in vivo* applications [e.g., when cardiac pacemakers can help to control abnormal heart rhythms (Lee et al., 2016)]. Electrical stimulation aims to elicit or modulate a functional response by providing a minimum injected charge through electrodes to cardiac cells and tissues.

Notably, due to the high optical transparency of graphene, graphene electrodes for the electrical stimulation of cells can be made transparent, allowing to combine both electrical and optical methods for stimulating and monitoring the functional activity of cardiomyocytes. The ability to stimulate cardiac cells while simultaneously monitoring their activity in real time using brightfield or fluorescent microscopy is critical in studies aimed at understanding the fundamental mechanisms of cardiovascular disorders, the search for novel cardiovascular drugs, and the evaluation of cardiotoxicity of any existing and novel drugs.

**FIGURE 8:**
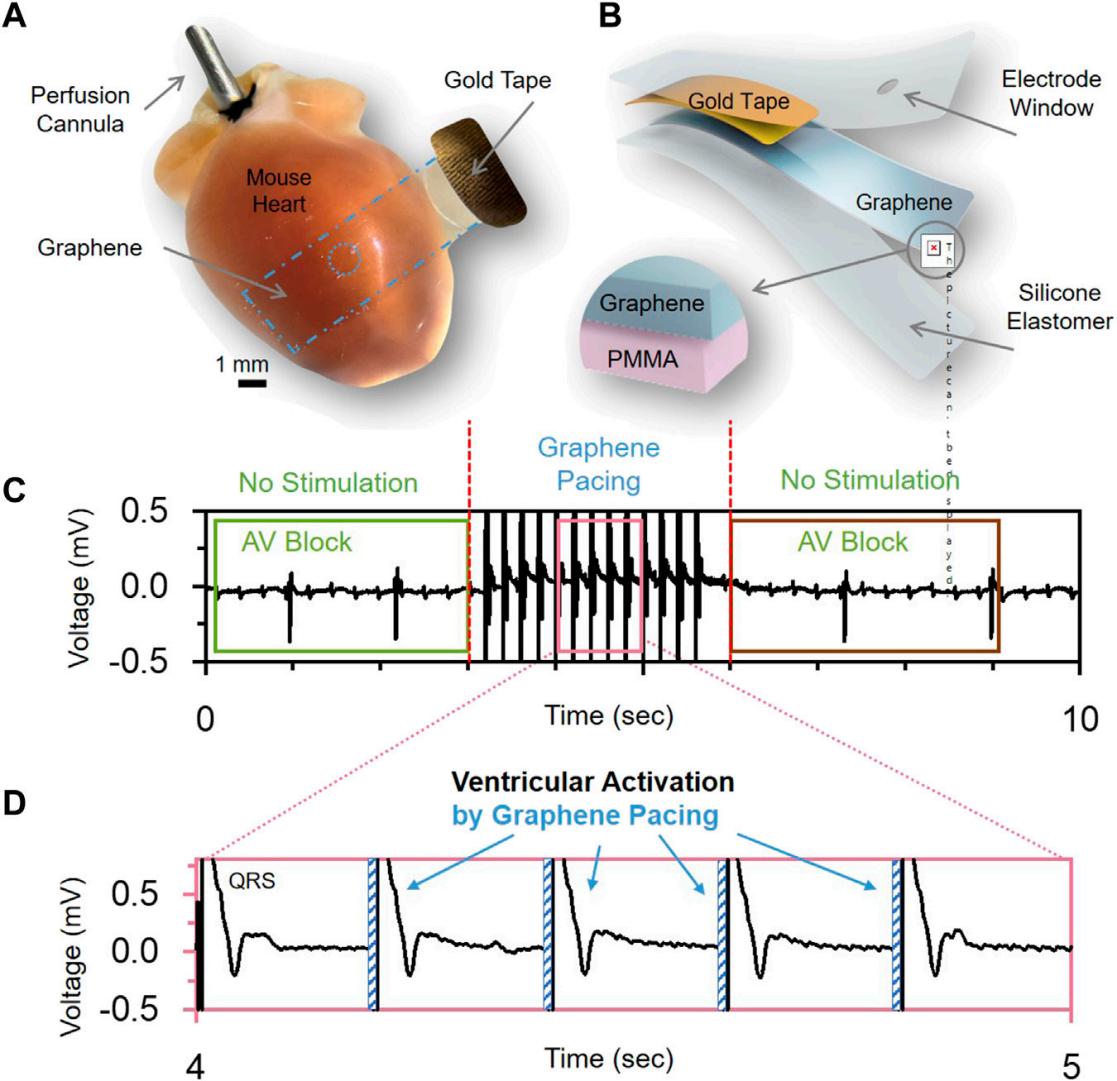
Graphene-based electrical actuators. **(A)**, Optical image and **(B)**, schematic of graphene electrodes used for pacing heart *in vivo*. **(C,D)**, demonstrate the ability to use the graphene electrodes for sensing (C) electrical activity of the heart with a disorder (AV Block) and ability to treat the disorder by applying electrical stimulation pulses **(D)**. Panels reproduced from open-access publication Lin et al. (2022), Wiley.

One area where graphene electrodes are expected to impact dramatically is chronic cardiac interfaces for cardiac pacemakers (Figures 8A, B). Graphene may help overcome current implantable devices’ limitations, such as rigidity, high inflammatory potential, and poor long-term stability in physiological environments (Kostarelos et al., 2017; Reina et al., 2017). Graphene electrodes have higher values of charge injection in comparison with common gold or platinum electrodes (Kostarelos et al., 2017) and provide better compatibility at the tissue-electrode interface, which would improve the efficiency of cardiac pacemakers by resolving problems such as scarring of cardiac tissues (Dvorak et al., 2012). Graphene electrodes can make it possible to pace hearts using lower electrical currents and decrease damage to cardiac tissues from repetitive electrical stimulation. Additional improvements in charge injection levels (up to tens of mC/cm^2^) can be achieved by combining graphene with other materials such as thermally evaporated cesium carbonate (Sanders et al., 2015), parylene-C (Apollo et al., 2015), platinum nanoparticles (Lu et al., 2018), or multi-stacking of several graphene monolayers (Park et al., 2016).

Several studies developed hybrid graphene-containing cell substrates that, in addition to offering an excellent topological microenvironment for cardiomyocyte development and maturation (Savchenko et al., 2021), can provide electrical stimulation capabilities during cell culturing.

Hitscherich et al. (2018) fabricated electrospun nanofibrous scaffolds containing graphene nanoparticles embedded into poly(caprolactone) (PCL). During electrical stimulation, graphene nanoparticles act as local conductive sites, distributing the external electrical field throughout the PCL scaffolds. The presence of graphene in these electroactive scaffolds and added electrical stimulation capabilities were synergistic in providing beneficial effects on the morphology and function of murine ESC-derived cardiomyocytes.

Li et al. (2021) developed hybrid cell substrates containing using reduced graphene oxide, polydopamine, and GelMA hydrogels. These substrates provided the opportunity to electrically stimulate cardiac microtissues resulting in a more functional and mature myocardium layer.

Lin et al. (2022) used GET-electrodes to perform cardiac actuating by connecting them to a cardiac stimulator. Cardiac pacing was performed using pulses of 0.5–2 ms in duration, resulting in a faster heart rate. Due to their transparency, these GET-electrodes were successfully used in optical mapping studies in a paced Langendorff-perfused mouse heart model when the changes in electrical activity and calcium transients were monitored using fluorescent voltage- and calcium-sensitive indicators.

The mGET-arrays (Lin et al., 2022) described earlier were used to treat atrioventricular block (AV block) in an *in vivo* rat model by providing reversible ventricular pacing at the rate of 300 BPM (Lin et al., 2022) (Figures 8C, D **)**. This is the first time when actuating graphene electrodes were used to successfully treat a life-threatening heart rhythm disorder.

### 3.2 Optical stimulation

Graphene exhibits remarkable optoelectronic properties, including the ability to efficiently convert light into electricity via a hot-carrier multiplication process on a femtosecond timescale (Gabor et al., 2011; Tielrooij et al., 2013; Johannsen et al., 2014). In graphene, light produces “hot” ballistic electrons (Gabor et al., 2011) that transfer their energy through a very efficient carrier–carrier scattering process, leading to multiple hot-carrier generation over a wide range of light frequencies. The mean free path of photogenerated “hot” ballistic electrons can be up to 1 µm (Novoselov et al., 2004), allowing the enhanced flexibility in spatial positioning of optoelectronic graphene biointerfaces near cells. These properties provide the foundation for graphene biointerfaces for optical stimulation of cells (Savchenko et al., 2018).

**FIGURE 9:**
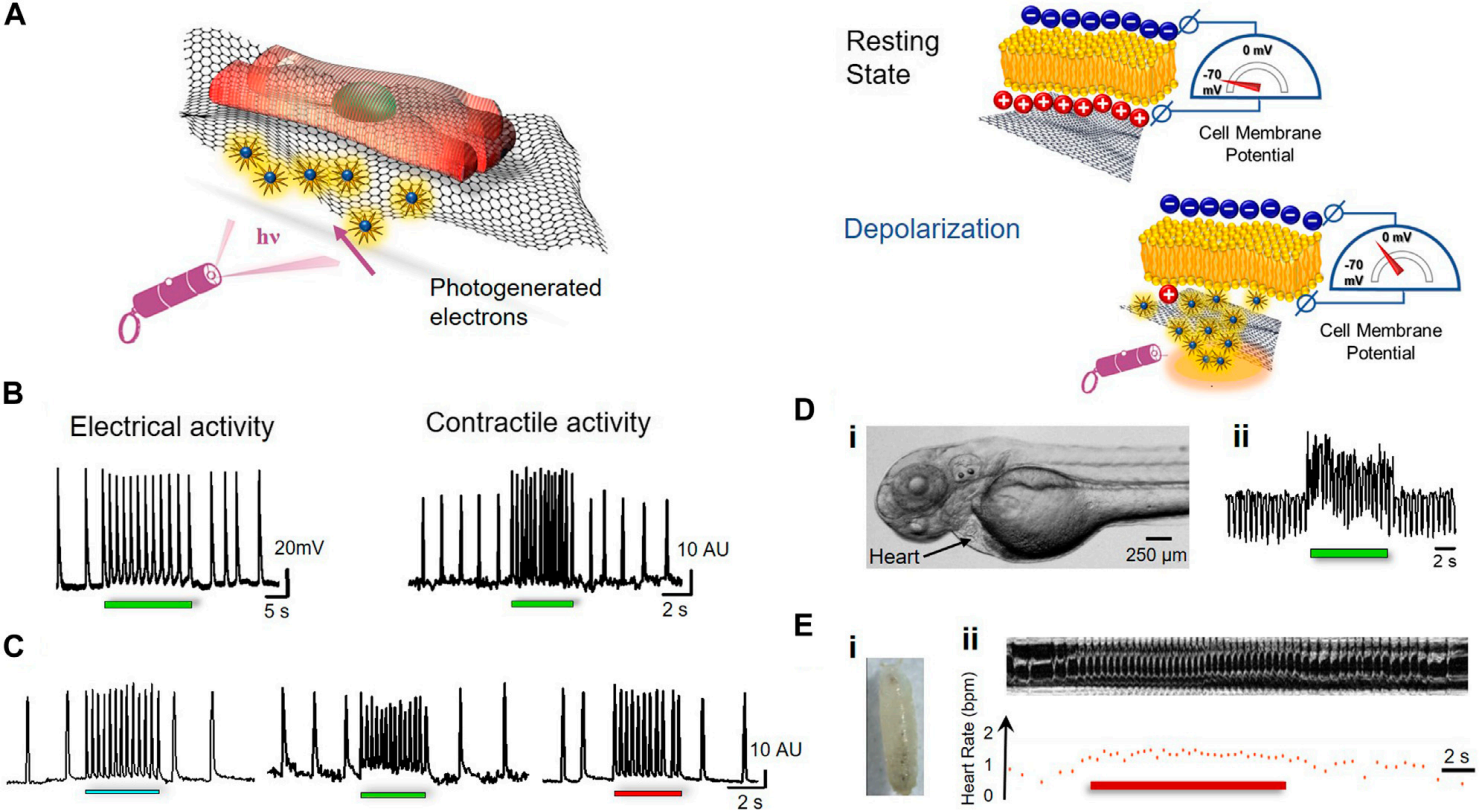
Graphene-based actuators. **(A)**, Working principle of optical actuating using the GraMOS platform, **(B)**, Light-induced changes in electrical activity (left) and contractile activity (right) of human induced pluripotent stem cell-derived cardiomyocytes, **(C)**, Broadband absorption of graphene allows using different wavelengths of light to modulate contractions of cardiomyocytes. **(Di)**, Zebrafish embryo treated with graphene; **(Dii)**, Representative heart contraction trace demonstrating GraMOS effects in graphene-treated zebrafish embryos; **(Ei)**, Drosophila Melanogaster pupa treated with graphene; **(Eii)**, Representative optical coherent microscopy M-mode trace (top) and corresponding heart rate plot (bottom) during GraMOS in graphene-treated pupa. Panels reproduced from open-access publications: **(B–D)**, (Savchenko et al., 2018), American Association for the Advancement of Science.

During graphene-mediated optical stimulation (GraMOS) (Figure 9A), photo-generated electrons from graphene (Tiberj et al., 2013) can change the cell membrane potential by displacing cations near the graphene/cell membrane interface due to the capacitive coupling effect (Freitag et al., 2013) between the cell membrane and graphene surface. Thus, GraMOS biointerfaces can provide optical stimulation of cells via an external light-generated electric field that interacts with the transmembrane field gradient in a physiological manner. This process first leads to membrane depolarization, then to activation of voltage-gated ion channels, and, finally, to action potential generation, calcium influx, and contractions of cardiomyocytes GraMOS was shown to elicit fast and reversible changes in the contraction rates of spontaneously contracting cardiomyocytes (Figure 9B), initiate the contractions in quiescent cardiomyocytes, or stop the contractions by inducing significant depolarization leading to inactivation of voltage-gated sodium channels.

GraMOS effects are optoelectronic rather than thermal in nature because graphene is a highly efficient light-to-electricity converter (Gabor et al., 2011; Tielrooij et al., 2013; Johannsen et al., 2014). Due to zero band-gap and strong electron-electron interactions in graphene, photogenerated electrons are poorly coupled to the graphene surface and preferentially distribute their energy to multiple secondary electrons rather than produce lattice heating (Gabor et al., 2011; Tielrooij et al., 2013). As a result, the light energy is delivered to “hot” electrons, while the graphene lattice remains “cold.” The experimental data further confirm that light-induced heat-mediated effects are unlikely during GraMOS, because 1) a light intensity required for GraMOS is ∼1,000 times lower than required for thermal effects (Jenkins et al., 2010; Shapiro et al., 2012; Thompson et al., 2014), 2) the surface temperature of optoelectronic graphene biointerfaces is unaffected during light exposure required for GraMOS (Savchenko et al., 2018), and 3) GraMOS triggers fast steady-state responses in cardiomyocytes, which is drastically different from gradual temperature-driven changes in the cell membrane potential (Shapiro et al., 2012) produced by heating. In contrast to a transparent optoelectronic GraMOS platform, a photothermal 3DFG platform absorbs almost all incident light and requires extremely high-intensity light to operate (Rastogi et al., 2020b).

The GraMOS platform has numerous advantages. The capacitive mechanism of GraMOS is more physiological than the Faradic mechanism of electrical stimulation (Merrill et al., 2005; Ghezzi et al., 2011), and does not lead to cellular damage often associated with electrical stimulation. In addition, GraMOS can be achieved using a wide range of light wavelengths (Figure 9C), because photon absorption in graphene is nearly constant in the range of 300–2,500 nm (Mak et al., 2008; Nair et al., 2008).

GraMOS also works in *in vivo* settings, including zebrafish (Savchenko et al., 2018) and *Drosophila Melanogaster* (Ugur et al., 2016), well-established animal models for biomedicine and drug discovery. After dispersible graphene flakes were injected into zebrafish embryos at 3 days post-fertilization, changes in heart contractions of graphene-treated zebrafish in response to light (wavelength 480 nm) illumination occurred fast (Figure 9D), and were dependent on the concentration of graphene biointerfaces. A similar phenomenon was observed in *Drosophila* (Matt et al., 2023) where graphene was injected into the heart tube of a wild-type early pupal fly (Figure 9E). Here, illumination of the heart tube using two wavelengths of light (470 and 617 nm) resulted in the increase in the heart rate, and was dependent on the illumination intensity. These results suggest that graphene-based actuators have the potential to enable optical cardiac pacemaking in the future.

## 4 Future perspectives

There is an impressive lineup of various types of graphene-based cardiac sensors and actuators taking advantage of a combination of unique properties of graphene and utilizing different mechanisms of action. At present, the development of these systems is still at the exploratory stage, when, in some cases, testing new ideas and initiating a pioneering study are prioritized over technical studies aimed at ensuring that these systems work properly rather than just work. Excitement stemming from new capabilities that graphene can bring into the area of cardiac biosensors and actuators is temporary and fully understandable. Currently, the focus has already begun shifting into the engineering and practical validation stage that will take advantage of all exploratory studies (including the ones reviewed here) to accelerate the advancement of cardiac sensors and actuators from a lab bench to patients.

The unique properties of graphene have already made this nanomaterial a practically indispensable element in the emergent bioengineering systems for cardiac biosensing and actuation. Future developments in this area will be fueled by new graphene-related discoveries and will be expected to revolutionize biosensing and actuating systems and help us to be healthier and live longer. A potential example for future applications is co-growing graphene nanomaterials with cellular organoids in order to use them for next-generation cardiac transplants, as was recently shown possible with the brain (Wilson et al., 2022). To translate the technology into commercially available products, it is essential to solve the main engineering challenge that holds for not only graphene but any ultra-soft bioelectronics systems: interconnects. The interconnects are essential to delivering power in, and information out of the transducers, which actually administer a large range of conditions on said interconnects. On one side, they must be ultimately thin but strong to have a tight connection to the graphene, flexible and highly conductive and elastic in the middle, but strong and firm on the other hand in order to connect to the outer world.

Finally, besides graphene, there are many other 2D materials that hold similar optical and mechanical properties but also provide new electrical functionalities (Bhimanapati et al., 2015; Akinwande et al., 2017). For example, a whole cohort of 2D materials is predicted to be piezoelectric (Cui et al., 2018; Huo et al., 2022; Sherrell et al., 2022; Bruno et al., 2023), which might provide a unique solution for harvesting energy right from the mechanical movement of the heart and using it to power the electrical transducers. Finally, one may draw onto the recent trend of developing artificial biohybrids that, in some examples, can also be made ultra-flexible, soft, and biocompatible (Kireev et al. (2022a)). These biohybrids can then provide the internal closed-loop “decision-making” element, sparing the need for external communication and creating a stand-alone self-powered and self-decisive system capable of sensing, interpreting, and stimulating the cardiac tissue.
